# Peribronchial Inflammatory Cell Assessment in COPD Lung Tissues

**DOI:** 10.1111/jcmm.70229

**Published:** 2024-11-24

**Authors:** Maëva A. Devilliers, Lynda Saber Cherif, Laure M. G. Petit, Nathalie Lalun, Arnaud Bonnomet, Anne Durlach, Gonzague Delepine, Myriam Polette, Jeanne‐Marie Perotin, Gaëtan Deslée, Valérian Dormoy

**Affiliations:** ^1^ University of Reims Champagne‐Ardenne (URCA), Inserm UMR‐S 1250, SFR Cap‐Santé Reims France; ^2^ PICT Platform, University of Reims Champagne‐Ardenne (URCA) Reims France; ^3^ Department of Biopathology University Hospital of Reims Reims France; ^4^ Department of Thoracic Surgery University Hospital of Reims Reims France; ^5^ Department of Respiratory Diseases University Hospital of Reims Reims France

**Keywords:** COPD, inflammation and airway remodelling, lung histology, MGG

Chronic obstructive pulmonary disease (COPD) is an inflammatory disease characterised by bronchial, bronchiolar and alveolar remodelling underlying inflammatory processes [1]. In addition to small airway alterations in COPD, the dedifferentiation of the bronchial epithelium appears to be a hallmark of the disease [2, 3]. Besides, inflammatory cell anomalies have been described in COPD, including altered cell counts in bronchoalveolar lavage fluids [4], or peribronchiolar inflammatory cell recruitment such as B cells and lymphoid follicles [5]. Studies investigating the relationship between the presence of inflammatory cells and COPD‐associated remodelling at the bronchial level are scarce. In this study, we compared the inflammatory cell counts (lymphocyte, neutrophil, basophil and eosinophil) in peribronchial region below the bronchial epithelium obtained from COPD and non‐COPD patients.

We selected 23 stable mild/moderate COPD patients and 11 non‐COPD patients (Table 1) treated by lung resection (FEV_1_ (%predicted): 76.96 ± 3.32 vs. 101.46 ± 3.93, *p* < 0.001; FEV1/FVC: 60.48 ± 1.25 vs. 78.18 ± 1.09, *p* < 0.001). We excluded severe and very severe COPD patients because we focused here on the immune cell recruitment that may occur early in the pathogenesis. Patients were matched for age (66.70 ± 2.33 vs. 66.82 ± 2.11 years, respectively, *p* = 0.974), sex (ratio F/M: 5/18 vs. 1/10, *p* = 0.380), body mass index (BMI, 24.26 ± 0.90 vs. 26.03 ± 1.63 kg/m^2^, *p* = 0.315), smoking status (ratio smokers/ex‐smokers: 9/14 vs. 5/6, *p* = 0.736) and pack‐years of smoking (45.09 [20–74] vs. 39.18 [7–90], *p* = 0.423) to rule out the influence of common confounding factors. Exclusion criteria included all other acute or chronic respiratory diseases.

**TABLE 1:** Subject characteristics.

*n*	COPD	Non‐COPD	*p*
	23	11	
*Demography and clinical characteristics*			
Age (years)	66.7 ± 2.33	66.82 ± 2.11	0.974
Sex—F/M	5/18	1/10	0.380
BMI (kg/m^2^)	24.26 ± 0.90	26.03 ± 1.63	0.315
Smokers/ex‐smokers	9/14	5/6	0.736
Pack‐years	45.09 [20–74]	39.18 [7–90]	0.423
*Functional and biological characteristics*			
FEV_1_ (% predicted)	76.96 ± 3.32	101.5 ± 3.93	**0.0001** **
FEV_1_/FVC	60.48 ± 1.25	78.18 ± 1.09	**2.4×10^–10^** **

*Note:* Values are means ± standard deviation, medians [interquartile ranges] or absolute numbers (percentages). Percentages given are derived from those subjects with valid data. *p* is in bold when it is < 0.05.

** *p* < 0.001.

Histochemistry stainings (May‐Grünwald‐Giemsa, MGG) were performed on formalin‐fixed paraffin‐embedded (FFPE) lung tissues (*n* = 34). After dewaxing and rehydratation, slides were stained with Jenner Stain Solution 50% (26114‐01, CliniSciences) for 6 min followed by Giemsa Stain Solution 6% (GGS500, CliniSciences) staining for 30 min. Then, slides were dehydrated using increasing ethanol gradients (95% and 100%) and xylene solution before mounting. Microscopic slides were acquired on a slide scanner (VS120, Olympus, Tokyo, Japan) equipped with a 60X oil immersion objective (NA 1.35, extended focus) with the VS120‐AW acquisition software (Olympus v2.9, Tokyo, Japan). Lymphocytes, neutrophils, basophils and eosinophils were visually identified and counted based on their histological examination by trained and experienced histopathologists using QuPath software, Belfast, UK. The neutrophils were also immunostained with elastase (MAB916471) to confirm their identity (data not shown). The inflammatory cells were counted in peribronchial regions, between the epithelium and the smooth muscle cell layer (this area was set as a reference), as well as in the epithelium. Epithelial remodelling features including epithelial thickness, basal cell counts (p63 positive), Ki67‐positive cells, ciliated surface, MUC5ac‐, MUC5b‐, CC10‐secreting cells and primary ciliated cells (Arl13b‐positive non‐differentiated cells) were quantified as previously described [6].

Statistical analysis was performed using GraphPad Prism 5 (GraphPad Software Inc.). Data were presented as median (range) for skewed distributions. Comparisons used the Student *t*‐test (two‐tailed) and correlations used the Pearson test, both for parametric data. Statistical significance was determined as *p* < 0.05.

The first novelty of our study is the experimental approach resorting to MGG staining on FFPE lung tissues to distinguish the major inflammatory cells on one single microscopic slide. MGG staining allows a fast quantitative analysis of eosinophil, neutrophil, basophil and lymphocyte counts in lung tissues (Figure 1a). No differential accumulation of eosinophils (43.08 ± 81.83 cells/mm^2^ vs. 18.56 ± 24.52 cells/mm^2^, *p* = 0.341), neutrophils (78.98 ± 58.64 cells/mm^2^ vs. 67.88 ± 61.14 cells/mm^2^, *p* = 0.614), basophils (75.98 ± 79.37 cells/mm^2^ vs. 46.82 ± 37.70 cells/mm^2^, *p* = 0.258) and lymphocytes (122.55 ± 136.03 cells/mm^2^ vs. 102.59 ± 61.25 cells/mm^2^, *p* = 0.647) was identified in mild/moderate COPD patients in comparison with non‐COPD patients (Figure 1b). The same observation was made considering only the smoking status on the whole cohort or in the smokers vs. ex‐smokers or considering intraepithelial localisation (data not shown). An intriguing association was unveiled showing higher BMI associated with higher eosinophils (*r* ^2^ = 0.245, *p* = 0.031) and neutrophils (*r* ^2^ = 0.231, *p* = 0.037) in COPD patients only.

**FIGURE 1:**
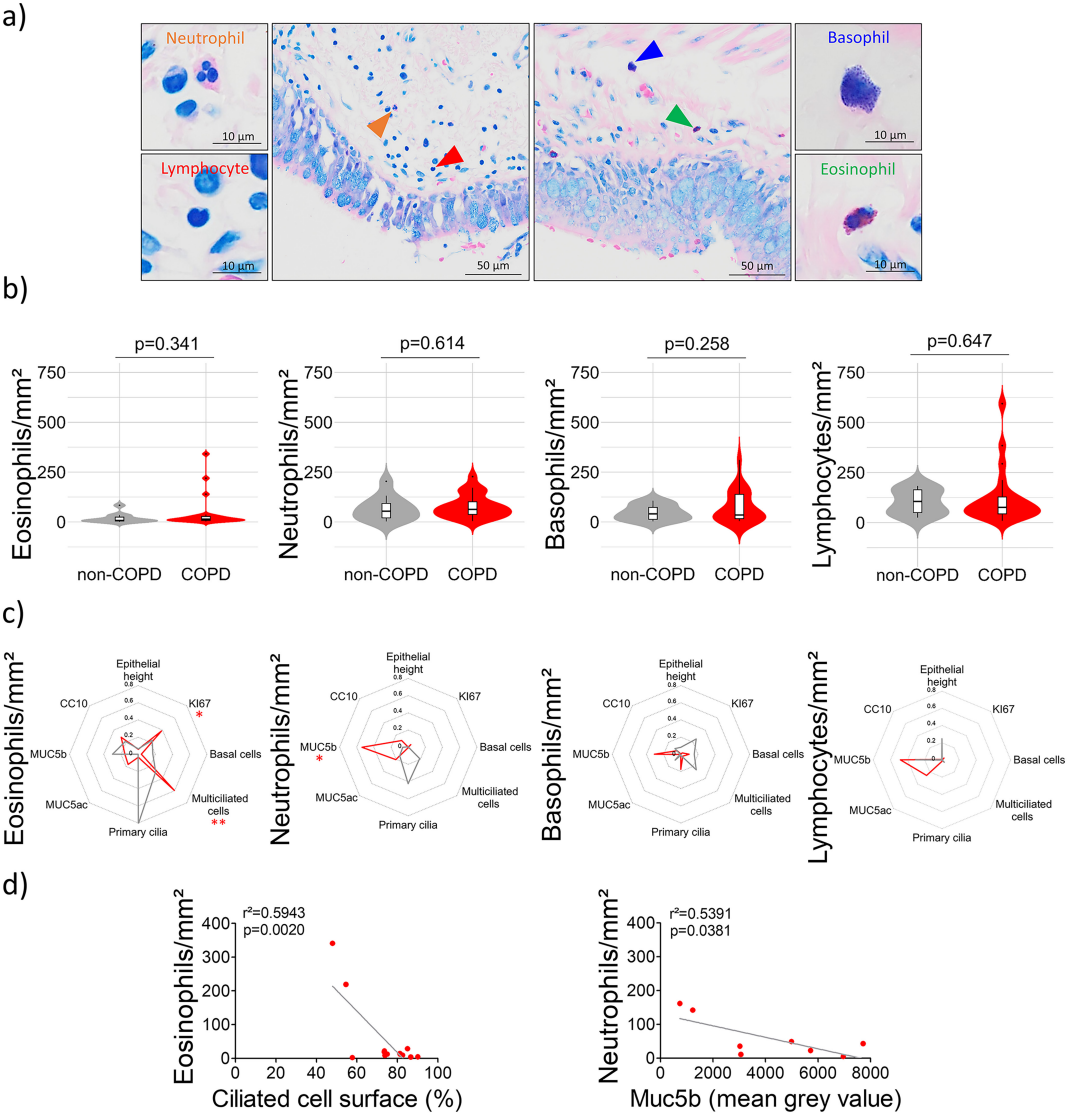
Quantitative analysis of peribronchial neutrophils, lymphocytes, basophils and eosinophils in COPD lung tissues. (a) Example of a microscopic acquisition showing peribronchial accumulation of neutrophils (orange arrow), lymphocytes (red arrow), basophils (blue arrow) and eosinophils (green arrow) in an MGG‐stained FFPE lung resection from a COPD patient. (b) Violin plots showing eosinophil, basophil, neutrophil and lymphocyte counts in the peribronchial regions of COPD (*n* = 23) and non‐COPD patients (*n* = 11). The markers for the median and the box indicating the interquartile range are shown in black. (c) Radar charts displaying the square of the Pearson correlation *r* for the multivariate epithelial remodelling features in non‐COPD (grey lines, *n* = 4–8) and COPD patients (red lines, *n* = 8–13) regarding eosinophil, basophil, neutrophil and lymphocyte counts. *p < 0.05, **p < 0.01 in COPD patients. (d) Dot plots showing linear regressions for COPD patients (*n* = 8–11) regarding eosinophil and neutrophil counts for the ciliated cell surface and Muc5b.

Taking strength from our previous characterisations of bronchial epithelial remodelling features in COPD patients [3, 6, 7], we then tested the associations of peribronchial inflammatory cell accumulation with eight potential readouts of COPD‐induced epithelial alterations [epithelium height, proliferation, basal cell count, multiciliated cell surface, primary ciliated cells, mucous‐secreting cells (MUC5ac and MUC5b) and CC10‐secreting cells]. Interestingly, in COPD patients only, there was an association between high basal cell proliferation and a high eosinophil count (*r* ^2^ = 0.382, *p* = 0.032), a decrease in ciliated cell surface and a high eosinophil count (*r* ^2^ = 0.594, *p* = 0.002) and an increase in MUC5b‐secreting cells and low neutrophil count (*r* ^2^ = 0.539, *p* = 0.038) (Figure 1c,d).

Altogether, we demonstrate the feasibility of performing MGG stainings on FFPE lung tissues. This experimental approach is readily available worldwide in diagnostic laboratories and allows a quick morpho‐histological characterisation of inflammatory cells in tissues. Consistent with our findings, the bronchial wall does not appear as a crucial site for immune cell infiltration but the loss of distal patterning in COPD prompted us to quantitatively analyse the presence of immune cells in bronchi. Interestingly, we found an association of eosinophil and neutrophil counts with bronchial epithelial remodelling features in COPD patients as documented in asthma [8, 9]. Our findings ideally complement previous observations obtained through haematoxylin and eosin stain, Luna stain, or immunohistochemical analysis with antibodies (elastase, ECP, BB1) on FFPE lung tissues mainly focused on small airways in severe COPD patients [10, 11, 12, 13]. The epithelial bronchial cellular composition directly influences mucociliary clearance orchestrated by multiciliated and mucin‐secreting cells, partly responsible for the recruitment and the activation of immune cell populations in the lumen and the wall of the airways. Here, we evidenced that an accumulation of bronchial eosinophils and a loss of bronchial neutrophils signed an epithelial remodelling characterised by a reduction of the epithelial ciliated surface and an imbalance of mucin production (ratio Muc5ac/Muc5b). This is in line with the understanding that COPD physiopathology relies on structural and inflammatory alterations. Future investigations are requested to address the cellular and molecular mechanisms fuelling and connecting both processes.

This study has a number of limitations. We did not include severe/very severe COPD patients where inflammatory cell accumulation may increase in the connective tissues of the airways. Although MGG staining is easy to implement, it should be supplemented with biomarkers distinguishing the large varieties of B cells, T cells or specific differentiation statuses of the granulocytes. Finally, we did not evaluate the presence of macrophages or dendritic cells that are also highly relevant to COPD pathophysiology.

In summary, our study highlights an absence of differential abundance of peribronchial lymphocytes, neutrophils, basophils and eosinophils in mild/moderate smokers/ex‐smokers COPD patients, and a correlation of eosinophil and neutrophil counts with bronchial basal cell proliferation, ciliated cells and mucous‐secreting cells. The next step will aim for the automatisation of the microscopic cell quantification, and the development of in vitro approaches to test the association between airway epithelial cell differentiation (from basal cells to multiciliated and mucous‐secreting cells) and eosinophils or neutrophils as evidenced, for example, in the context of the alarmins and their biological regulations [14].

## Author Contributions

**Maëva A. Devilliers:** formal analysis (lead), investigation (lead), writing – original draft (lead), writing – review and editing (lead). **Lynda Saber Cherif:** investigation (supporting), writing – review and editing (supporting). **Laure M. G. Petit:** investigation (supporting), writing – review and editing (supporting). **Nathalie Lalun:** investigation (supporting), methodology (supporting), writing – review and editing (supporting). **Arnaud Bonnomet:** formal analysis (supporting), investigation (supporting), methodology (supporting), software (supporting), writing – review and editing (supporting). **Anne Durlach:** data curation (equal), formal analysis (supporting), investigation (supporting), methodology (supporting), resources (supporting), writing – review and editing (supporting). **Gonzague Delepine:** data curation (supporting), resources (equal), writing – review and editing (supporting). **Myriam Polette:** data curation (supporting), formal analysis (supporting), writing – review and editing (supporting). **Jeanne‐Marie Perotin:** data curation (equal), investigation (supporting), methodology (supporting), resources (equal), writing – original draft (supporting), writing – review and editing (supporting). **Gaëtan Deslée:** data curation (equal), formal analysis (supporting), funding acquisition (supporting), investigation (supporting), resources (supporting), writing – original draft (supporting), writing – review and editing (supporting). **Valérian Dormoy:** conceptualization (lead), data curation (equal), formal analysis (equal), funding acquisition (lead), investigation (equal), methodology (lead), project administration (lead), resources (equal), supervision (lead), validation (equal), visualization (equal), writing – original draft (lead), writing – review and editing (lead).

## Ethics Statement

The study was conducted in accordance with the Declaration of Helsinki and the use of human tissues was authorised with the written consent of patients (Biological Collection DC‐2012‐1583, IRB 00003888 Inserm 21‐775).

## Conflicts of Interest

Gaëtan Deslée reports lecture honoraria from Chiesi and AstraZeneca; outside the submitted work. Valérian Dormoy reports lecture honoraria from Chiesi and AstraZeneca; outside the submitted work. There are no further conflicting interests to disclose.

## Data Availability

The data that support the findings of this study are available from the corresponding author upon reasonable request.
